# Acute Pain Management in Peripheral Artery Disease: A Holistic, Beyond-Opioids, Individualized Multimodal Approach

**DOI:** 10.4274/TJAR.2024.241657

**Published:** 2024-12-16

**Authors:** Maria P. Ntalouka, Athanasios Chatzis, Petroula Nana, Konstantinos Spanos, Metaxia Bareka, Miltiadis Matsagkas, Eleni Arnaoutoglou

**Affiliations:** 1 University of Thessaly Faculty of Medicine; Larissa University General Hospital, Department of Anaesthesiology, Larissa, Greece; 2 University of Thessaly Faculty of Medicine; Larissa University General Hospital, Department of Vascular Surgery, Larissa, Greece

**Keywords:** Acute pain, multimodal treatment, pain, pain management, peripheral artery disease

## Abstract

Peripheral artery disease (PAD) is quite prevalent, and its incidence will increase with aging of population. Pain is a key diagnostic feature of symptomatic PAD and has been linked to disease progression and poor quality of life. Symptom improvement is of utmost importance in PAD; therefore, optimal and comprehensive pain therapy is mandatory. However, the management of acute pain in PAD remains challenging due to the lack of high-quality evidence, the complex pathophysiological mechanisms of pain, and the high comorbidity of patients. On the other hand, inadequate pain control leads to several pathophysiological deviations, such as the aggravated neuroendocrine stress response, which may be detrimental in patients with PAD. Experts suggest that the management of acute pain in patients with vascular diseases should be oriented toward the underlying pathophysiological mechanisms of each modality and should follow a multifactorial approach. Although the exact pain pathways in PAD are still poorly understood and more probably multifactorial, they may be key to an effective, individualized, patient-centered, multimodal pain strategy. The aim of this review was to provide a holistic, beyond-opioids, individualized multimodal pain approach for patients with PAD.

**Main Points**

• Management of patients with acute pain due to peripheral artery disease (PAD) is challenging due to high comorbidity and complex pathophysiology.

• Multimodal pain management is the cornerstone of optimal treatment of acute PAD-induced pain.

• The neuropathic component of pain is of utmost importance.

• Opioids are the gold standard treatment for severe pain.

• Multimodal analgesia, invasive techniques, and non-pharmaceutical interventions are effective and safe approaches for the management of acute pain due to PAD.

## Introduction

Peripheral artery disease (PAD) is defined as the clinical spectrum of chronic vascular insufficiency due to widespread arterial atherosclerosis that predominantly affects the segments of the lower limb (aortoiliac, femoropopliteal, and infrapopliteal).^1, 2, 3, 4^ Although its incidence varies, more than 200 million patients worldwide are affected by PAD.^1, 3^ The infrapopliteal arterial form is the most prevalent, with a reported incidence of up to 20%.^1, 3^ As the population continues to age, the incidence of PAD will increase, severely affecting the quality of life and longevity of patients. However, because PAD is often asymptomatic, it may remain underdiagnosed, underrecognized, or undertreated.

Pain is a key diagnostic feature of symptomatic PAD, and it has a major impact on quality of life and everyday function. Worsening pain has been linked to disease progression.^4, 5, 6, 7, 8^ Hence, optimal and comprehensive pain treatment in patients with PAD is mandatory. However, pain management in this population proves to be challenging due to the lack of high-quality evidence, the underlying complex pathophysiological mechanisms of pain, and the high comorbidity-including ischemic heart disease, impaired renal function, and diabetes mellitus-which further complicate the provision of therapeutic pain interventions.^5^ On the other hand, inadequate pain control leads to several pathophysiological deviations such as the aggravated neuroendocrine stress response and the activation of the autonomic nervous system (ANS), which may prove to be detrimental in patients suffering from PAD.^5^ Although the exact pain pathways and mechanisms in symptomatic PAD are multifactorial and still poorly understood, they are considered to be valuable assets that will lead to an effective, individualized, and patient-centered, multimodal pain strategy.

The first part of this review presents a short overview of the clinical manifestations, pathophysiology, and etiology of pain in patients with PAD. We then provide a holistic, beyond-opioids, individualized multimodal pain approach for acute pain in patients with PAD based on the relevant pain pathways.

### Pain Manifestations of PAD

Although PAD is usually asymptomatic in the early stages, up to 50% of patients progress to symptomatic disease in which pain prevails. The key diagnostic characteristics of painful PAD are chronic and gradually worsening pain frequency and intensity, with exacerbations of acute pain. Pain characteristics have been linked to disease progression, while clinical manifestations vary from intermittent claudication (IC) to critical limb ischemia (CLI). Of note, CLI without treatment can cause tissue and limb loss.^4, 5, 7, 8, 9^

IC, otherwise known as stable PAD, presents as cramp-like pain in the muscle group distal to the atherosclerotic lesions. Buttock and thigh claudication indicate aortoiliac segment disease, whereas calf claudication indicates femoropopliteal segment disease. IC pain follows a characteristic pattern: (i) pain is absent at rest, as there is adequate blood supply for the tissues; (ii) is triggered by exercise and develops progressively due to muscle ischemia; and (iii) is eased by a short period of rest. In the vast majority of patients, IC pain recurs with the same pattern and at a similar walking distance.^1, 2, 5^ However, some patients may not present with the classic features of IC, but they may fall under the “leg pain/carry on” or “leg pain on exertion and rest” symptoms. Leg pain that occurs with exertion but does not force the patient to stop walking is known as “leg pain/carry on”, while pain that is consistently triggered by activity but may be present at rest is known as “leg pain on exertion and rest”.^2^

On the other hand, CLI is characterized by severe pain at rest, which is worse on elevation and only relieved by dependency.^1, 2, 5^ Pain is the result of inadequate tissue perfusion and is often more intense at night, due to the absence of the effects of gravity on blood flow. Thus, it is relieved by hanging the lower extremity off the bed or by standing up and walking. Rest pain in CLI may be accompanied by tissue loss ranging from ulcers to frank necrosis and gangrene, which may lead to major amputation and resultant stump pain, phantom limb pain, or post-amputation mechanical back pain.^1, 2, 4, 5, 7, 8^

### Pathophysiology and Etiology of Pain in PAD

The pathophysiology of pain in PAD is multifactorial, complex, and not fully understood. Based on the latest proposed classification, PAD pain may be nociceptive, neuropathic, or mixed, where nociceptive and neuropathic elements coexist (Table 1).^4, 5, 7, 8, 9, 10^ Identification of the primary pathophysiological pathway in each stage of PAD is fundamental for the constitution of an appropriate treatment strategy (Table 2).^4, 5, 7, 8, 10, 11^

Nociceptive pain is mediated through nociceptive receptors, which are located in the outer and middle layers of the wall of large and medium-sized arteries. These receptors may be activated by dilation or dissection, whereas the painful stimulus is often further enhanced by stimulation of the ANS fibers that cover large vessels, such as the aorta. Moreover, in patients with PAD, nociceptive pain further escalates because of the destruction of tissues by chronic inflammation, which in turn activates the somatosensory nervous system.^5, 9^ This surge of inflammatory mediators, including cytokines and chemokines, triggers nociceptors and subsequently downregulates their threshold, a phenomenon known as peripheral sensitization. This state of increased responsiveness is responsible for the activation of the threshold of pain pathways from non-painful or lower threshold stimuli and for the perceived aggravated response to noxious stimulation.^5^

Neuropathic pain is the result of a lesion or disease of the somatosensory nervous system. This may have an impact on the function or structure of the somatosensory nervous system, leading to sensory loss and an increased responsiveness to noxious and innocuous stimuli. Neuropathic pain is a critical component of CLI and indicates the long-term nature of the underlying disease. Experts suggest that alterations in ion channels, G-protein-coupled receptors, neurotransmitters, and central activation constitute the main pathophysiological components of neuropathic pain.^5^ Moving on to mixed pain, both advanced IC and CLI, where both neuropathic and nociceptive pain elements coexist with severe chronic inflammation, constitute a typical example.^5, 9^ In addition, patients with PAD may suffer from mixed pain after lower limb amputation. Following limp amputation, an interaction of the sympathetic nervous system with the first-order sensory neuron is established, which leads to central sensitization, which is the increased responsiveness of the brain and spinal cord nociceptors to normal or below-normal intensity of afferent stimulus. The aforementioned changes, with subsequent modifications of vascular network reactivity, indicate the existence of central/complex regional pain syndromes (CRPS), which play a key role in phantom limb pain.^5^

It should be highlighted that in 2016, the term nociplastic pain was defined as “pain that arises from altered nociception despite no clear evidence of actual or threatened tissue damage that causes peripheral nociceptors activation or evidence of disease or lesion of the somatosensory system causing the pain”.^12^ According to the latest literature, this fairly new concept of pain appears in chronic pain conditions. From a pathophysiological point of view, three main mechanisms have been recognized: supraspinal, spinal, and peripheral mechanisms. However, no study has indicated the implication of oncoplastic pain in any stage of PAD.^12^

### Proposed Stepwise Pain Management for PAD

According to experts, managing the multifaceted nature of pain in PAD is challenging and hence requires a multifactorial approach. Of note, in addition to the proposed pain management strategy, optimal management of PAD seems to be of paramount importance for the adequate alleviation of pain. Nevertheless, when the exacerbations of chronic pain are considered, treatment of the neuropathic element of pain is considered to be rather essential.^5, 6^

Based on the current literature, it is recommended that optimal pain management should be individualized according to the patient, his/her comorbidities, the underlying pathophysiology of the pain, and the respective clinical entity (PAD). Holistic multifactorial analgesia based on pharmaceutical agents, invasive techniques, and non-pharmacological methods appears to prevail because it targets several sites throughout the pain pathways, providing better analgesic effects.

### Pharmaceutical Pain Management

The model of Channels-Enzymes-Receptors Targeted Analgesia (CERTA), a multimodal pain strategy, is proposed for optimal pharmaceutical pain management.^2^ Based on CERTA, a pain treatment strategy is adopted according to the pathophysiological pathways of pain in each stage of PAD. This model utilizes a variety of analgesic agents, depending on pain pathways, in low doses in terms of maximum safety and therapeutic efficacy for each agent. CERTA intends to be a stepwise therapeutic intervention with the titration of several opioids and non-opioid analgesics as the intensity of pain increases. Tables 3, 4, 5 summarizes the proposed pharmaceutical pain management strategies according to pain intensity and the primary pathophysiological element.^10, 13, 14, 15, 16, 17, 18, 19^

It should be noted that the use of non-steroidal anti-inflammatory drugs (NSAIDs) in patients with PAD requires extreme vigilance because of the high probability that there might be several contraindications for administration, including comorbidities, bleeding predisposition, or active bleeding.^5, 6, 14, 17, 18^ Moreover, in contrast to intravenous or oral NSAIDs, topical or transdermal NSAIDs achieve analgesia through local infiltration and subsequently increase the concentration of the drug up to 4-7 times in the target tissues compared with plasma concentrations. Pharmaceutical forms intended for topical use are considered ideal for patients with impaired renal function or elderly patients prone to elevated plasma concentrations of the drug, as well as for patients with multiple comorbidities, such as patients with gastric ulcer and cardiovascular diseases, in which the use of oral NSAIDs is contraindicated.^14, 17, 18^

### Invasive Techniques

If the above therapeutic interventions fail and the pain intensity remains high (numerical rating scale-*NRS* score or visual analog scale score>7), the use of invasive techniques in the form of peripheral nerve block (PNB) is mainly recommended in patients with CRPS.^15^ Moreover, PNB attenuate the sympathetic tone and produce significant vasodilation, two important features in PAD-related ischemic pain. For patients with stump and phantom limb pain after amputation, PNB with the addition of clonidine to the local anaesthetic solution remains the gold standard. In patients undergoing below the knee amputation, the block of the sciatic nerve is sufficient, in contrast to the one above the knee amputation where the block of the femoral nerve is necessary.^5^

### Non-pharmaceutical Pain Management

The implementation of non-pharmaceutical interventions is strongly recommended in parallel with pharmaceutical measures and always depends on the patient’s age, developmental status, prevailing conditions, and severity of the current clinical condition (Table 6).^14, 19^

### Intermittent Pneumatic Compression

Although high-quality data are lacking, intermittent pneumatic compression (IPC) could serve as a safe and effective alternative for patients with non-operable PAD in an attempt to alleviate the symptoms, including pain, of PAD and CLI. It appears that IPC can reduce pain intensity and increase pain-free walking distance in patients who are not suitable candidates for open or endovascular surgical treatment and in those who require palliative care. The main idea behind this favorable profile of IPC is that implementation of IPC can improve venous outflow and arterial flow and can increase the shear stress and release of nitric oxide by the endothelium, leading to vasodilation and decreased peripheral resistance.^20, 21^

### Lifestyle Changes

### Nutrition and Diet Therapy

Although high-quality data are still missing, it seems that the Mediterranean diet, along with nuts and polyunsaturated fats, may be associated with a lower incidence of PAD, while it may improve blood flow and reduce pain in patients with established PAD.^22^ Overall, adherence to a healthy diet, rich in antioxidants, in an attempt to reduce chronic inflammation, oxidative stress, and endothelial dysfunction has been associated with improved outcomes in PAD.^22^ Moreover, it has been reported that oral antioxidants such as vitamin E, vitamin C, beta-carotene, ginkgo biloba, cocoa, and flavonoids may lead to longer pain-free walking distances in patients suffering from chronic PAD.^22, 23^ However, there is a paucity of data regarding their value in the acute phase of the disease.

### Smoking Cessation

Smoking can increase the levels of potent vasoconstricting peptides, such as endothelin-1, for approximately 15 minutes.^22^ Long-term increased endothelin-1 levels can reduce muscle blood flow and compromise vasodilation.^23^ Thus, smoking cessation is associated with a decreased risk of progression from PAD to CLI, improved walking ability, and decreased claudication symptoms.^22, 23^ However, a direct association between smoking cessation and ischemic pain exacerbation has not been established.^22^

### Supervised Exercise Therapy

Exercise training, including aerobic and strength training, as tolerated with gradually increased physical activity or exercise to near-maximal claudication levels, has been proven to be beneficial to ischemic pain management due to enhanced perfusion, muscle oxygen extraction capacity, regulation of vessels, and vascular endothelium function.^22^ Compared with home-based training, supervised exercise therapy (SET) is a more effective strategy for the management of symptomatic PAD.^22^ SET is defined as 30-60 min of treadmill or track walking to the point of pain, followed by rest. SET walking programs are the recommended first-line therapy for claudication.^22^ Self-directed walking programs are recommended as second-line treatment when SET is not applicable. According to the results of the ERASE trial, SET combined with revascularization in patients with aortoiliac or femoropopliteal PAD demonstrated increased maximum walking distance, pain-free walking distance, and better quality of life.^22, 23^ However, SET is contraindicated for patients with ischemic pain at rest, and there is a paucity of data regarding its value in the acute phase of the disease. Lastly, regarding high- or low-intensity exercises, there is no available evidence to support their value in terms of better outcome.^22^

**Figure 1:**
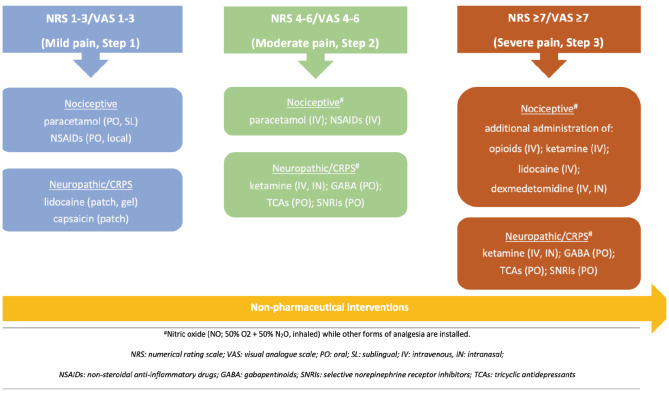
Algorithm for stepwise pain management in PAD. PAD, peripheral artery disease.

Figure 1 summarizes the proposed algorithm for a stepwise and multimodal pain approach for PAD.

## Conclusion

PAD is prevalent, and pain is a key diagnostic feature of symptomatic PAD. Inadequate pain control may be detrimental to patients with PAD. Hence, optimal and comprehensive pain therapy is mandatory. However, the management of acute pain in PAD remains challenging. An individualized, patient-centered, multimodal pain strategy based on the underlying pathophysiological mechanisms of each stage of PAD, including lifestyle modifications, is key to a holistic and effective pain approach for patients suffering from PAD.

## Figures and Tables

**Table 1. Pathophysiological Classification of Pain in Patients with PAD:** 

**Primary pathophysiological mechanism of pain**	**PAD stage**
Nociceptive	Ischemia (acute ischemia or early-stage PAD) Ischemia (end-stage PAD) Stump pain Mechanical back pain after limb amputation
Neuropathic	Ischemia (end-stage PAD) Stump pain
Mixed	Ischemia (end-stage PAD) CRPS: stump pain, phantom limb pain, mechanical back pain after limb

PAD, peripheral artery disease; CRPS, complex regional pain syndrome

**Table 2. Etiology of Acute Pain in Patients with PAD:** 

**Acute pain**	
Acute lower extremity ischemia	Atherosclerotic plaque rupture
**Acute exacerbations of chronic pain**	
IC, CLI	Atherosclerosis with progressive disease deterioration
**Limb amputation**	
Stump pain	Exposure of large and different tissue surfaces to a multitude of nociceptors under strong noxious stimulation
Phantom limb pain	Central sensitization
Mechanical back pain	Exacerbation of pre-existing conditions, prolonged recumbency, and early stages of prosthesis use

PAD, peripheral arterial disease; IC, intermittent claudication; CLI, critical limb ischemia

**Table 3. Pharmaceutical Pain Management in Mild Pain (NRS 1-3/VAS 1-3, Step 1):** 

Nociceptive	Paracetamol (1 g) Paracetamol SL 0.5gr x2 NSAIDs: ibuprofen, 400 mg; naproxen, 500 mg; diclofenac, 50 mg; celecoxib, 200 mg NSAIDs: diclofenac topical gel 1% (maximum dose: 2 gr upper extremities, 4 gr lower extremities), solution 1.5% (maximum dose: 40 drops), transdermal patch 1.3% (1 patch)
Neuropathic Mixed/CRPS	Lidocaine: transdermal patch 4-5% (up to 3 patches), lidocaine 2.5% + prilocaine 2.5% gel (up to 20 gr in 200 cm^2^) Capsaicin: transdermal patch 3.75-8% (up to 4 patches)

NRS, numerical rating scale; VAS, visual analogue scale; PO, oral administration; SL, sublingual administration; NSAIDs, non-steroidal anti-inflammatory drugs; CRPS, complex regional pain syndrome

**Table 4. Pharmaceutical Pain Management for Moderate Pain (NRS 4-6/VAS 4-6#, Step 2):** 

Nociceptive	Paracetamol (1 g) NSAIDs: ibuprofen IV 400-800 mg, diclofenac NSAIDs: diclofenac topical gel 1% (maximum dose: 2 gr upper extremities, 4 gr lower extremities), solution 1.5% (maximum dose: 40 drops), transdermal patch 1.3% (1 patch)
Neuropathic Mixed/CRPS	Ketamine: IV 0.1-0.3 mg kg^-1^ (single dose in 10-15 minutes) or IV 0.15 mg kg^-1^ h^-1^ (continuous infusion), or IN 0.7-1 mg kg^-1^ Gabapetin 50 mg or pregabalin 25 mg (GABA) Duloxetine PO 30 mg (SNRIs) Amitriptyline PO 10-25 mg (TCAs)

^#^Nitric oxide (NO; 50% O_2_ + 50% N_2_O, inhaled), while other forms of analgesia are installed.

NRS, numerical rating scale; VAS, visual analogue scale; IV, intravenous administration; PO, oral administration; NSAIDs, non-steroidal anti-inflammatory drugs; CRPS, complex regional pain syndrome; GABA, gabapentinoids; SNRIs, selective norepinephrine receptor inhibitors; TCAs, tricyclic antidepressants

**Table 5. Pharmaceutical Pain Management in Patients with Severe Pain (NRS ≥7/VAS ≥7#, Step 3):** 

Nociceptive	In addition to Step 2, the following steps are repeated: Morphine IV 0.05-0.1 mg kg^-1^ Fentanyl IV 0.5-1.0 µg kg^-1^ Ketamine: IV 0.1-0.3 mg/kg (single dose in 10-15 minutes) or IV 0.15 mg kg^-1 ^h^-1^ (continuous infusion), or IN 0.7-1 mg kg^-1^ Lidocaine IV 1-2 mg kg^-1^ (single dose over 10 minutes, 200 mg maximum dose) or IV 0.5-3 mg kg^-1 ^h^-1^ (continuous infusion, based on ideal body weight, 200 mg maximum dose) Dexmetatomidine IV 0.5-1.0 µg kg^-1 ^(loading dose in 10 minutes) or IV 0.5-2 µg kg^-1 ^h^-1^ (continuous infusion) or IN 1-2 µg kg^-1^
Neuropathic Mixed/CRPS	In addition to Step 2, the following steps are repeated: MgSO_4_ IV 70 mg kg^-1^ (in 4 hours with an average flow of 25 mL hr) Dexamethasone IV 8 mg with simultaneous administration of 250 mL 10% mannitol Morphine IV 0.05-0.1 mg kg^-1^ Fentanyl IV 0.5-1.0 µg kg^-1^ Lidocaine IV 1-2 mg kg^-1^ (single dose over 10 minutes, 200 mg maximum dose) or IV 0.5-3 mg kg^-1 ^h^-1^ (continuous infusion, based on ideal body weight, 200 mg maximum dose) Dexmetatomidine IV 0.5-1.0 µg kg^-1^ (loading dose in 10 minutes) or IV 0.5-2 µg kg^-1 ^h^-1^ (continuous infusion) or IN 1-2 µg kg^-1^

^#^Nitric oxide (NO; 50% O_2_ + 50% N_2_O, inhaled) while other forms of analgesia are installed.

NRS, numerical rating scale; VAS, visual analogue scale; IV, intravenous administration; ΙΝ, intranasal administration

**Table 6. Non-pharmaceutical Interventions:** 

Adequate time available for history taking and patient interaction	Music therapy
Reassurance and attentive listening	Aromatherapy
Empathy	Discussion with a psychologist or cognitive therapy
Eye contact	Massage
Use of wheelchair or wheeled bed	Acupuncture
Resting body position and/or resting of the suffering limb	Transcutaneous electrical nerve stimulation
Tools for distraction: television, tablet applications, virtual reality, and book reading	Infiltration and/or massage of trigger points
